# Tuning Pore Size in Graphene in the Angstrom Regime for Highly Selective Ion–Ion Separation

**DOI:** 10.1021/acsnano.3c11068

**Published:** 2024-02-06

**Authors:** Kangning Zhao, Wan-Chi Lee, Mojtaba Rezaei, Heng-Yu Chi, Shaoxian Li, Luis Francisco Villalobos, Kuang-Jung Hsu, Yuyang Zhang, Feng-Chao Wang, Kumar Varoon Agrawal

**Affiliations:** † Laboratory of Advanced Separations (LAS), École Polytechnique Fédérale de Lausanne (EPFL), Sion, CH-1950 Switzerland; ‡ CAS Key Laboratory of Mechanical Behavior and Design of Materials, Department of Modern Mechanics, University of Science and Technology of China, Hefei 230027, China

**Keywords:** single layer graphene, partial dehydration, angstrom-scale pore, ion selectivity, pore size distribution, selective ion transport

## Abstract

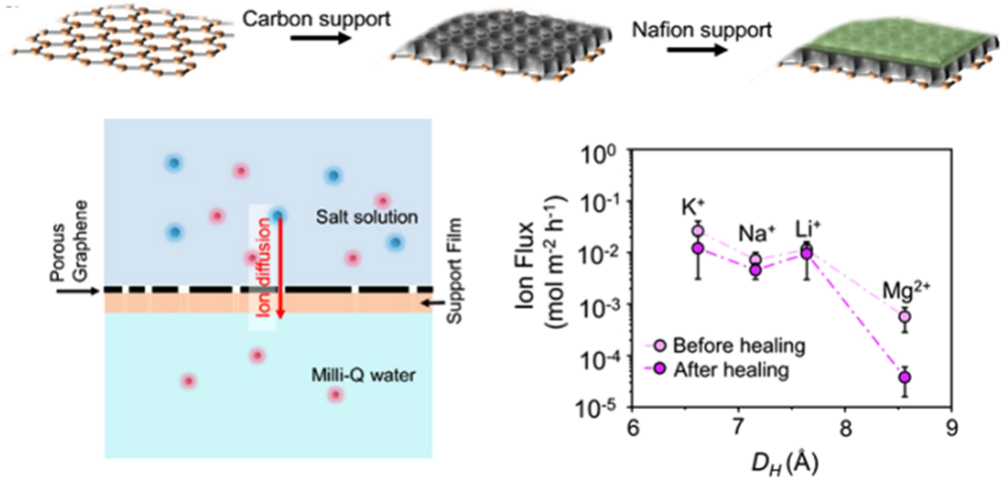

Zero-dimensional pores spanning only a few angstroms in size in two-dimensional materials such as graphene are some of the most promising systems for designing ion–ion selective membranes. However, the key challenge in the field is that so far a crack-free macroscopic graphene membrane for ion–ion separation has not been realized. Further, methods to tune the pores in the Å-regime to achieve a large ion–ion selectivity from the graphene pore have not been realized. Herein, we report an Å-scale pore size tuning tool for single layer graphene, which incorporates a high density of ion–ion selective pores between 3.5 and 8.5 Å while minimizing the nonselective pores above 10 Å. These pores impose a strong confinement for ions, which results in extremely high selectivity from centimeter-scale porous graphene between monovalent and bivalent ions and near complete blockage of ions with the hydration diameter, *D*_*H*_, greater than 9.0 Å. The ion diffusion study reveals the presence of an energy barrier corresponding to partial dehydration of ions with the barrier increasing with *D*_*H*_. We observe a reversal of K^+^/Li^+^ selectivity at elevated temperature and attribute this to the relative size of the dehydrated ions. These results underscore the promise of porous two-dimensional materials for solute–solute separation when Å-scale pores can be incorporated in a precise manner.

## Introduction

Porous graphene hosting Å-scaled pores has emerged as a highly promising material as the selective layer for membrane-based separation. It is regarded as a model system for such a study because solute confinement can be tuned by regulating the size of the pore,^1−3^ allowing a confinement-dependent mass transport study.^4^ However, a precise pore size control in a two-dimensional material lattice in the subnanometer range has proven to be challenging since the pore nucleation is usually accompanied by pore expansion and pore–pore coalescence and crack formation in the film leading to a limited resolution in solute–solute separation.

Solute confinement inside zero-dimensional pores spanning only a few Å in size results in mass transport which strongly deviates from the classical continuum transport.^5−9^ When the confinement is strong, i.e., when the pore size is comparable to that of the solute, the mass transport rate of a solute is dependent on the change in its solvation properties upon confinement. For hydrated ions, their partitioning from bulk water to the confined environment and *vice versa* is determined by the coordination environment of solute in the bulk and within the pore.^10−12^ The mass transfer rate of ions has been shown to be determined by the size difference between the pore and the ion hydration diameter in the bulk (*D*_*H*_),^13,14^ ionic charge,^15^ their tendency to dehydrate, i.e., the interaction with the coordinated solvent,^16,17^ hydration shell polarization,^18^ and ion core size.^19^ Based on this, pores in carbon and boron nitride nanotubes,^20−23^ graphene oxide,^24,25^ MoS_2_,^26^ metal–organic frameworks,^27^ nanoporous polymers,^28,29^ covalent organic framework,^30,31^ and atomically flat van der Waals (vdW) slits^19^ have been used to demonstrate the size exclusion as well as the Donnan exclusion effects. Å-scale biological protein channels, e.g., a potassium channel, exploit the confinement effect to perfection where a tight coordination of bare K^+^ ions with carbonyl ligands in the channel promotes the desolvation of the hydrated K^+^, which results in a large preference for K^+^ against other ions with a similar *D*_*H*_.^32,33^

Recently, it was shown that atomically flat vdW slits with a gap of 3.4 Å reject all ions except protons.^34,35^ It was also shown that 6.6–6.7 Å sized vdW slits yield only moderate selectivities between a small monovalent cation (K^+^, *D*_*H*_ = 6.6 Å) and a large cations such as Al^3+^ (*D*_*H*_ = 9.6 Å, selectivity of 4) and tetramethylammonium (*D*_*H*_ = 12.5 Å, selectivity of 50).^35^ It was also observed that when *D*_*H*_ is comparable to the gap, the diameter of the ion core, i.e., diameter without the hydration shell, influences ion mobility in confinement.^19^ Mobility of ions with larger cores was found to be suppressed. This was attributed to the preference of these ions to stay closer to the channel wall. This suppressed ion transport phenomenon is also observed in the one-dimensional channel-like carbon nanotube.^36^

Given that *D*_*H*_ values of the commonly studied monovalent salt ions (K^+^, Na^+^, Li^+^) are in the range of 6.6–7.6 Å, and common bivalent ions (Ca^2+^, Mg^2+^, Zn^2+^) are in the range of 8.2–8.6 Å, pores that are larger than 3.5 Å (i.e., larger than steric exclusion limit) but smaller than 8.5 Å should yield selectivity between these two sets of ions. The size range (3.5–8.5 Å) is also interesting because partial dehydration of ions is expected to play an important role here given that the confinement size is close to *D*_*H*_ for several common ions. For example, a recent molecular dynamics simulation predicted partial dehydration of the water shell of K^+^ for transport across a 4.2 Å-sized pore.^17^ While significant progress has been carried out in incorporating pores that are around 3–4 Å, or larger than 10 Å,^37,38^ limited progress has been made in incorporating pore size distribution around the size range of 4–10 Å, a range which is interesting for the separation of common salt ions.^39^

Herein, we use a combination of CO_2_ and CH_4_ to tune Å-scale pores in single-layer graphene to achieve a size distribution where pores smaller than 8.5 Å dominate. The chemistry is uniform, and a centimeter-scale graphene membrane could be prepared. Porous graphene could be suspended between two well-mixed reservoirs with one hosting water and the other hosting a solution of common inorganic salts, as widely discussed in the literature. Measuring the ion diffusion rate, we show that these pores yield high selectivities between ions based on the relative differences in *D*_*H*_ and resist the transport of ions with *D*_*H*_ larger than 9 Å. For example, H^+^/Fe^3+^ and H^+^/Al^3+^ selectivities were greater than 3 × 10^4^. K^+^/Fe^3+^ and K^+^/Al^3+^ selectivities approached 500. K^+^/Mg^2+^ selectivity over 300 could be achieved. We show that ion transport is accompanied by an energy barrier which increases with *D*_*H*_ (K^+^ < Na^+^ < Li^+^ < Mg^2+^). The presence of an energy barrier indicates the onset of partial dehydration of ions (reorganization through graphene lattice) for pore crossover which is confirmed by the molecular dynamics (MD) simulation. We observed that the trend for K^+^ and Li^+^ transport is reversed at and above 38 °C, which can be attributed to the smaller size of partially dehydrated Li^+^. These findings advance the prospects of differentiating similar-sized ions.

## Results

**Figure 1:**
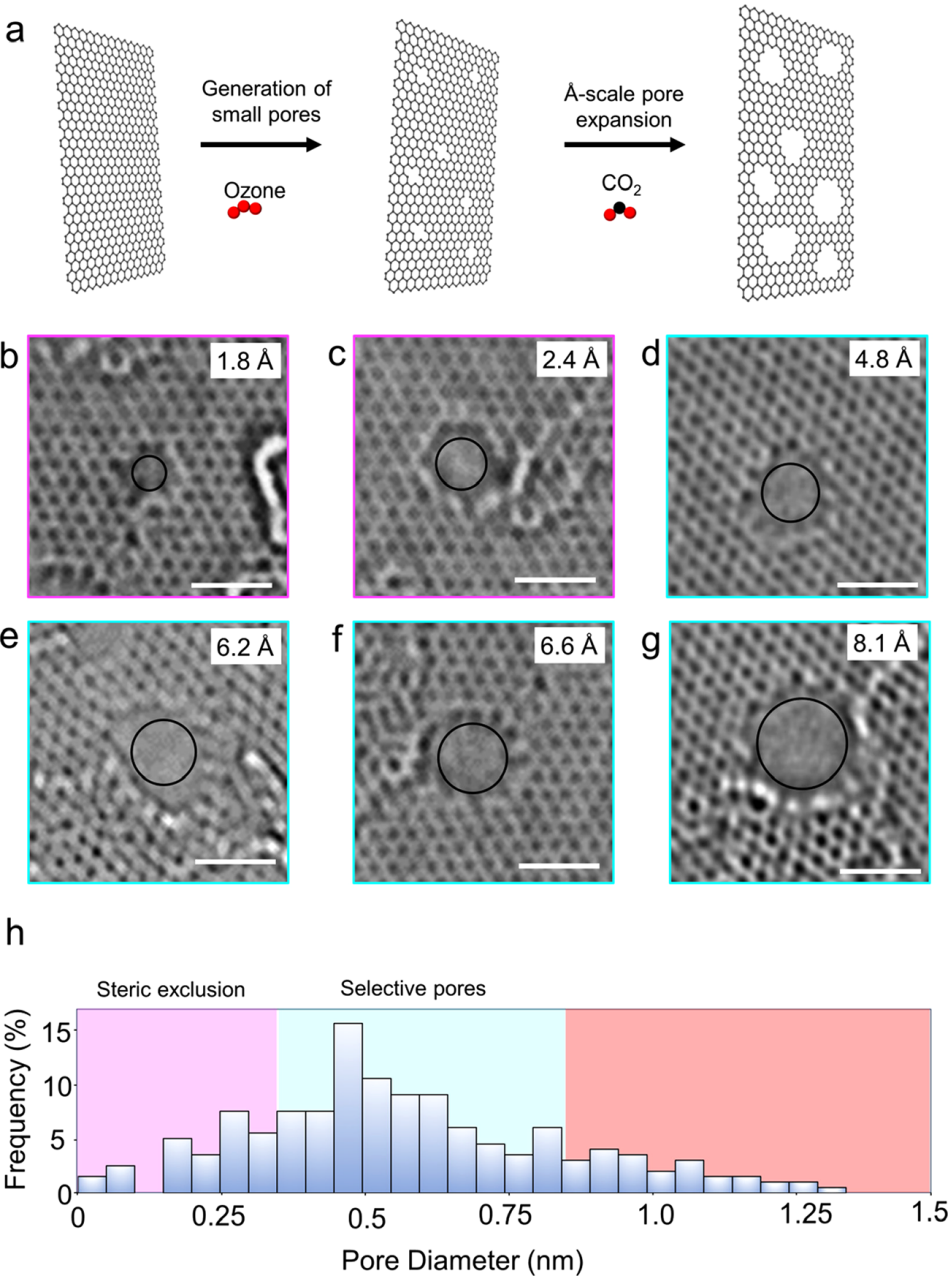
Generation of ion-selective pores in graphene. (a) Schematic illustration of controlled expansion of Å-scale pores in graphene. (b–g) AC-HRTEM images from PG_<8.5Å_ showing several pores with size ranging from 1.8–8.1 Å. Scale bars represent 1 nm. (h) Pore size distribution from PG_<8.5Å_.

Polycrystalline single-layer graphene film was synthesized on Cu foil by chemical vapor deposition (CVD). The dense lattice of graphene does not allow permeation of ions barring protons.^40,41^ The as-prepared graphene was of high quality and had a low density of intrinsic defects (*I*_*D*_), as confirmed by the Raman mapping data (*I*_*2D*_/*I*_*G*_ = 2.8 ± 0.4, *I*_*D*_/*I*_*G*_ = 0.05 ± 0.04, Figure S1). We refer to as-synthesized CVD graphene as “G_ID_” where *I_D_* refers to intrinsic defects. The graphene lattice can be postsynthetically modified to introduce pores. Gas-sieving pores with a size range of 3–4 Å have been successfully demonstrated by UV/O_3_,^42^ O_3_,^43^ and electron beam exposure.^9^ However, controlled expansion of these pores to a size below 10 Å has proven challenging. This is because of concomitant pore nucleation and expansion during extended etching, which promotes pore–pore coalescence. As a result, such efforts have led to size distribution where pores larger than 10 Å dominate^44,45^ (Figure S2). To resolve this, we used CO_2_ for pore expansion, which has an extremely high energy barrier for pore nucleation. The energy barrier for dissociative chemisorption of CO_2_ on the pristine graphitic lattice is large (∼5 eV)^46^ which prohibits reaction with the graphene basal plane. On the other hand, the energy barrier for pore expansion is moderate (2.7 eV) and can be surpassed with a pore expansion rate of ∼O (1 Å/min) for Å-scale pores.^47^ Motivated by this, we incorporated 3–4 Å pores in graphene^48^ by oxidation with O_3_ as reported before (referred to as “PG_3–4Å_” where PG refers to porous graphene), followed by pore expansion with CO_2_ for 5 min (Figure 1a).

Aberration-corrected high-resolution transmission electron microscopy (AC-HRTEM) of porous graphene and subsequent statistical size distribution data collected from approximately 200 pores confirmed that the majority of pores were smaller than 8.5 Å (Figure 1b–h, detailed analysis in Supplementary Note S1 and Figure S3). Based on this, we refer to PG prepared in this way as “PG_<8.5Å_”. The density of pores, including single vacancy defects expected to permeate protons, was consistent (2.2 × 10^12^ cm^–2^) before and after the CO_2_ expansion step. This confirmed that the CO_2_ expansion did not nucleate new pores. We note that AC-HRTEM data indicates a small fraction (8%) of pores with a size above 10 Å (Figure 1h). However, most of these pores are elongated (Figure S4), a sharp departure from rounded smaller pores (Figure 1b–g). These are created by the coalescence of nearby pores during the pore expansion by CO_2_. However, defect reorganization led coalescence during imaging by a 80 kV electron beam cannot be ruled out.

**Figure 2:**
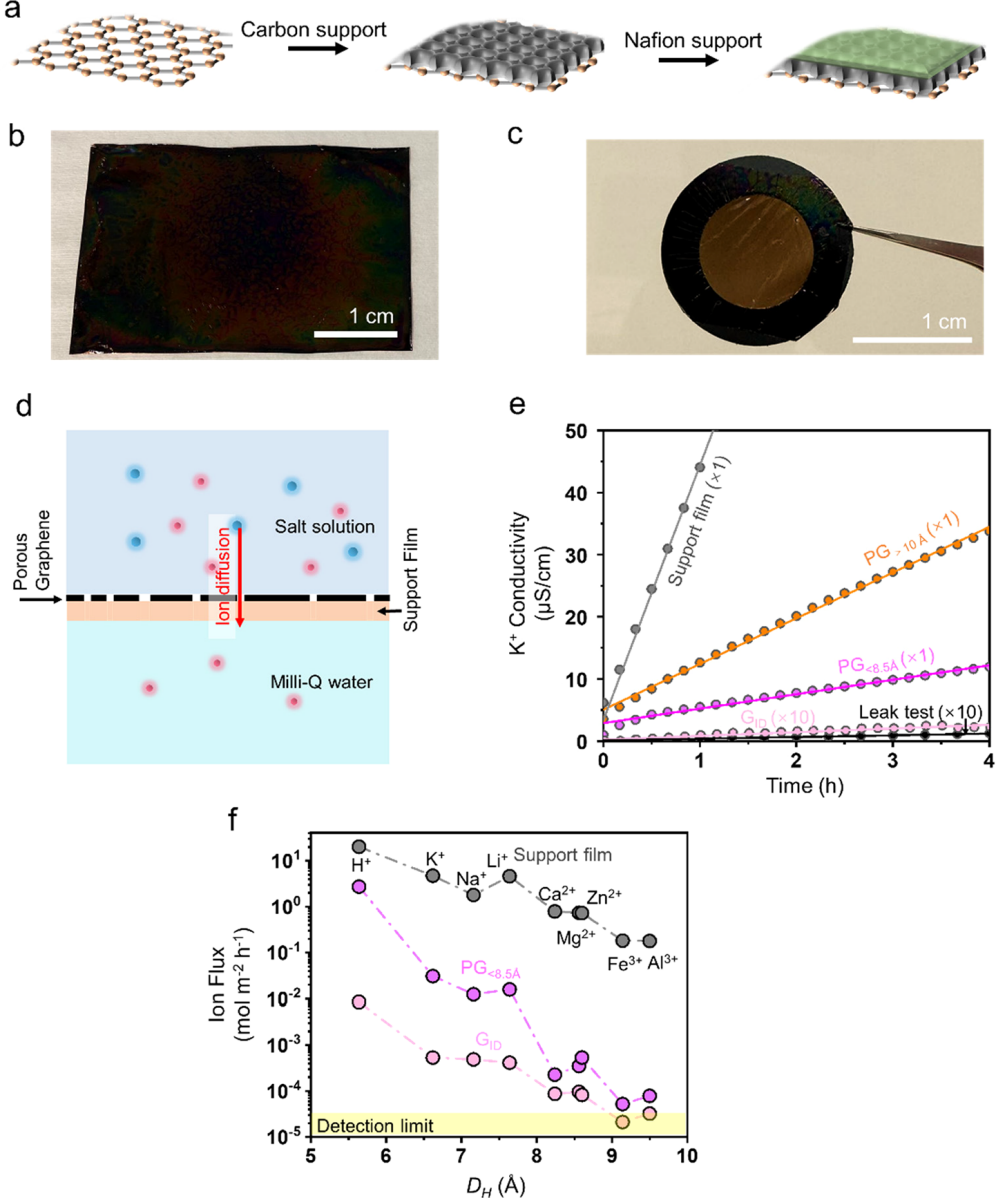
Ion–ion selective transport from porous graphene. (a) Schematics of the fabrication of the support film on top of graphene. (b) Picture of graphene reinforced with the support film, resting on a Cu foil (b) and suspended on an annular gasket (c). (d) Schematic of ion diffusion experiment across the porous graphene film. (e) Plot of K^+^ conductivity vs time from the support film, PG_>10Å_, PG_<8.5Å_, G_ID_, and an impermeable Kapton film. (f) Ion flux as a function of *D*_*H*_ for several mono-, di-, and trivalent ions across support film, PG_>10Å_, PG_<8.5Å_, and G_ID_. The error bars denote the standard deviation for three to five samples. The detection limit corresponds to the leak rate from the impermeable Kapton film.

To probe ion transport, we suspended graphene film on a porous carbon support hosting a porous lamellar microstructure with 20–30 nm sized pore openings^49^ (details in Experimental Section/Methods). The carbon support was 400 nm thick and was further mechanically reinforced with a 350 nm-thick Nafion film (Figure S5a,b) to improve handling and to minimize forward osmosis of water in the concentration-driven ion diffusion test (Supplementary Note S2 for details). The composite carbon/Nafion film is hereafter referred to as the “support film”. The support film was used as a mechanical reinforcement for graphene to assist crack-free transfer. We could prepare this film uniformly over a large area of graphene (Figure 2b) with good smoothness on both the Nafion and graphene sides (Figure S5e,f). This strategy allowed us to suspend graphene on an annular disk with an opening of 1 cm without any cracks or tears, while avoiding any other backing support layer (Figure 2c). The resulting suspended film was mechanically robust, exemplified by successful loading of weight (7 g) on top of the film (Figure S5c). Scanning electron microscopy and atomic force microscopy revealed a smooth and uniform surface of the film (Figure S5d-f).

An H-type diaphragm cell was used to probe concentration-gradient-driven ion diffusion. Graphene was sealed between the two reservoirs of the cell (Figure S6). The side of the cell contacting graphene was used as the feed side. It was filled with solutions of common inorganic chlorides with a concentration of 1 M. The other side (permeate side) was filled with Milli-Q water (Figure 2d). Both sides were stirred to avoid concentration polarization at the interface. A conductivity probe was inserted into the permeate side to monitor the ion concentration as a function of time. Inductively coupled plasma-optical emission spectrometry (ICP-OES) was used to correlate the change in solution conductivity to the change in ion concentration (Figures S7 and S8). A control test using an impermeable Kapton film and an aluminum tape revealed a negligible ion leak, confirming a good seal (Figure 2e). The support film led to a rapid linear increase in the concentration of the salt ions in the permeate side (Figure 2e). In contrast, for G_ID_, K^+^ transport was reduced by a factor of 10^4^ or higher, consistent with the low density of intrinsic defects in the as-synthesized graphene. This also confirmed that the graphene film was stable and blocked ion transport.

Next, we tested PG_<8.5Å_ where a discernible K^+^ flux was observed. The transport rate was 2 orders of magnitude smaller than that of the support film, indicating that graphene pores controlled the ion transport (Figure 2e). The fluxes for ions with *D*_*H*_ in the range of 6.5–7.6 Å (K^+^, Na^+^, Li^+^) were several folds larger than those of the ion with *D*_*H*_ larger than 8.6 Å (Mg^2+^, Figure 2f), yielding average K^+^/Mg^2+^, Li^+^/Mg^2+^, and Na^+^/Mg^2+^ selectivities of 46.0, 45.1, and 35.4, respectively. These selectivities are much larger than those based on bulk diffusivity in water (1.4, 0.7, and 0.9, respectively)^37^ or those from the support film (6.0, 5.9, and 2.3, respectively), confirming that the origin of the selectivity was ion confinement inside the pores of PG_<8.5Å_. This was further confirmed by loss of ion selectivity (Figure 2f) when the CO_2_ expansion time was deliberately increased where pores larger than 1 nm dominate (PG_>10Å_, Figure S9).

To further probe the confinement effect of PG_<8.5Å_, we carried out a transport study of several other ions (1 M concentration) including a smaller ion (proton) and larger ions with *D*_*H*_ up to 9.5 Å (Ca^2+^, Zn^2+^, Fe^3+^, Al^3+^). We observed extremely rapid transport of proton with a flux only one-tenth of that from the support film. This is not surprising because oxidation generated a high density of single-vacancy defects (Figure S10) which are permeable to protons but impermeable to other ions.^41^ The selectivity between the proton and the largest ions probed here (Al^3+^, Fe^3+^, *D*_*H*_ > 9 Å) was higher than 3 × 10^4^, indicating the exclusion of ions with *D*_*H*_ larger than 9 Å (Supplementary Note S2). Pores were stable in the environment of salt solution, thanks to the absence of pore-blocking hydrocarbon contaminants in the aqueous phase.^7,19^ During an extended period of testing (3 weeks), we observed stable ion flux and selectivities from a mixed equimolar feed (0.1 M KCl, NaCl, LiCl, and MgCl_2_, Figure S11).

**Figure 3:**
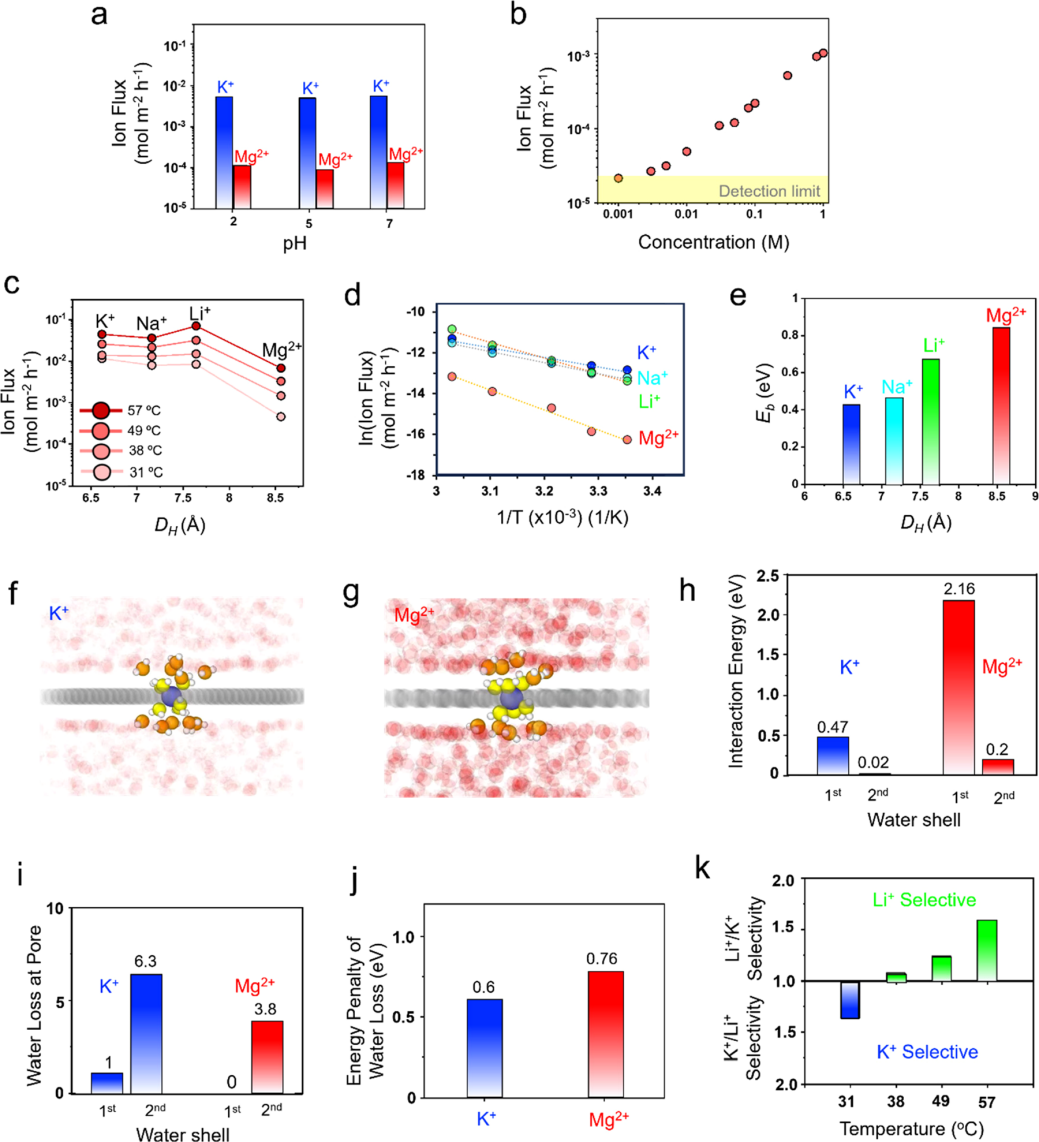
Partial dehydration of ions across Å-scale pores. pH-dependent (a) and concentration-dependent (b) K^+^ and Mg^2+^ transport from PG_<8.5Å_. (c) Diffusive ion flux from PG_<8.5Å_ as a function of temperature. (d) Arrhenius plot corresponding to the data in panel (c). (e) *E*_*b*_ extracted as per eq 1 as a function of *D*_*H*_. Snapshot of the partially dehydrated K^+^ ion (f) and Mg^2+^ ion (g) at the 6.1 Å-sized pore. (h) Interaction energy for K^+^ and Mg^2+^ ions with water molecules in the 1^st^ and 2^nd^ hydration shells. (i) Loss of the coordinated water molecules in the 1^st^ and 2^nd^ hydration shells for K^+^ and Mg^2+^ ions at the 6.1 Å-sized pore. (j) Energy penalty corresponding to the loss of the coordinated water as shown in panel (i). (k) K^+^/Li^+^ and Li^+^/K^+^ selectivities as a function of temperature.

While we observed the presence of the O-functional groups in PG_3–4Å_ (see high-resolution X-ray photoelectron spectroscopy (XPS) data in Figure S12 and Supplementary Note S3), pores in PG_<8.5Å_ were devoid of the functional groups. The C1s peak does not show any significant shoulder typically associated with C–O or C=O groups similar to pristine graphene and in contrast with PG_3–4Å_. This is because PG_3–4Å_ is prepared by oxidation by O_3_ where functional groups are generated on the graphene lattice. On the other hand, PG_<8.5Å_ is prepared by CO_2_-led pore expansion at 800 °C where O-functional groups are gasified.^43,48,50^ This is consistent with the literature where CO_2_ has been used to clean contaminants on the surface of graphene.^51^ The lack of a functional group on PG_<8.5Å_ indicates that pores were devoid of a significant charge. This is expected to limit the electrostatic interaction between the pore and the ions. Indeed, ion permeation data as a function of pH (2, 5, and 7) revealed that ion flux did not change significantly as a function of pH (Figure 3a). Concentration-dependent permeation of Mg^2+^ (10^–3^–1 M) did not show any saturation effect. The ion flux had a near-linear relationship with the concentration (Figure 3b). The ion selectivities from the mixed ion feed and single ion feed tests were similar (Figure S13), indicating that the competitive ion-sorption is not dominating the ion transport through graphene nanopores. Based on this, we conclude that the electrostatic interactions between ions and pores were limited.

To diffuse under the confined environment of the pores, ions are expected to partially dehydrate.^17,52,53^ Therefore, the ion arriving at the pore center should be in a transition state with a potential energy higher than that of the ions in the bulk. In this case, the ion flux can be described using the transition state theory and expressed in the Arrhenius form^11^ (eq 1):
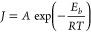
1where *J* is the ion flux, *A* is the pre-exponential factor, *R* is the universal gas constant, *T* is the temperature, and *E*_*b*_ is the energy barrier for the partition of an ion from the bulk (fully hydrated ion) to the center of the pore (partially dehydrated ion).

To understand whether a significant *E*_*b*_ is present for ion transport through PG_<8.5Å_, we carried out temperature-dependent diffusion tests (31, 38, 49, and 57 °C). The flux of all ions (K^+^, Na^+^, Li^+^, and Mg^2+^) increased with the temperature (Figure 3c). We could fit a modified version of eq 1 (ln *J* as a function of 1/*T*) with a high goodness of fit (R^2^ > 0.98) for all ions, confirming the presence of an energy barrier (Figure 3d). *E*_*b*_ increased for ions with a larger *D*_*H*_ (K^+^ < Na^+^ < Li^+^ < Mg^2+^, Figure 3e) from 0.42 eV for K^+^ to 0.84 eV for Mg^2+^. This energy barrier is likely due to an increasingly higher dehydration penalty for larger ions for their confinement in Å-scale zero-dimensional pores.

To understand the onset of dehydration, an MD simulation was carried out with porous graphene inserted in a simulation box separating two reservoirs of water (Figure 3f,g, Figure S14, details in Supplementary Note S4). We probed the hydration shells of K^+^ and Mg^2+^ ions in the bulk and when they are confined inside a pore. Subsequently, the interaction energies between the ion and water molecules from the first and second hydration shells were compared. The interaction of Mg^2+^ with its first and second hydration shell (−2.16 and −0.27 eV, respectively) and K^+^ with its first hydration shell (−0.47 eV) is quite strong. However, the interaction between K^+^ and a water molecule from its second hydration shell is quite weak (−0.02 eV, Figure 3h). This is consistent with the radial distribution function for both ions where the peak corresponding to the second shell of K^+^ is very broad. (Figure S15). This indicates that K^+^ will readily lose water from the second shell.

Next, by confining ions to the center of the graphene pore, we studied their hydration environment. Based on this, we extracted the coordination number of water molecules in the first and second hydration shells for K^+^ and Mg^2+^ as a function of the pore size (Figure S16). As the pore diameter decreases close to *D*_*H*_, ions progressively dehydrate and lose coordinated water. Therefore, the energy barrier associated with dehydration is the dominant effect for the Å-scale pores. Correspondingly, the loss in the coordination number in the first and second shells for ions placed inside a 6.1 Å pore, slightly smaller than *D*_*H*_ of K^+^, was extracted (Figure 3i). Based on the interaction energy with the water shells and the loss of the coordination number, we estimated the energy penalty associated with the water loss (Figure 3j). Indeed, the energy penalty for Mg^2+^ is much higher than that of K^+^, consistent with the experimental results.

Other important considerations besides ion dehydration include reorganization of the water shell where the entropic penalty of reorganization will also contribute to ion mobility across the pores. One of the implications of partial dehydration and reorganization of the hydration shell is that the transport rate would depend on the size and shape of the partially dehydrated ion and not *D*_*H*_. The Li^+^ ion has a smaller first hydration shell compared to that of K^+^, and therefore, upon losing its second hydration shell, Li^+^ is expected to cross zero-dimensional confinement of the pore with relative ease compared to the K^+^ ion.^18^ Indeed, we observe a faster Li^+^ transport compared to K^+^ at higher temperatures (≥38 °C) (Figure 3c) where the energy to surpass *E*_*b*_ becomes more accessible. The evolution of ion pair selectivity as a function of temperature clearly illustrates this (Figure 3k). At 31 °C, K^+^ transports faster with K^+^/Li^+^ selectivity of 1.4. At higher temperatures, this selectivity is reversed, and the Li^+^/K^+^ selectivity increases monotonically with temperature. The highest Li^+^/K^+^ selectivity was 1.6 at the highest temperature that we probed (57 °C).

**Figure 4:**
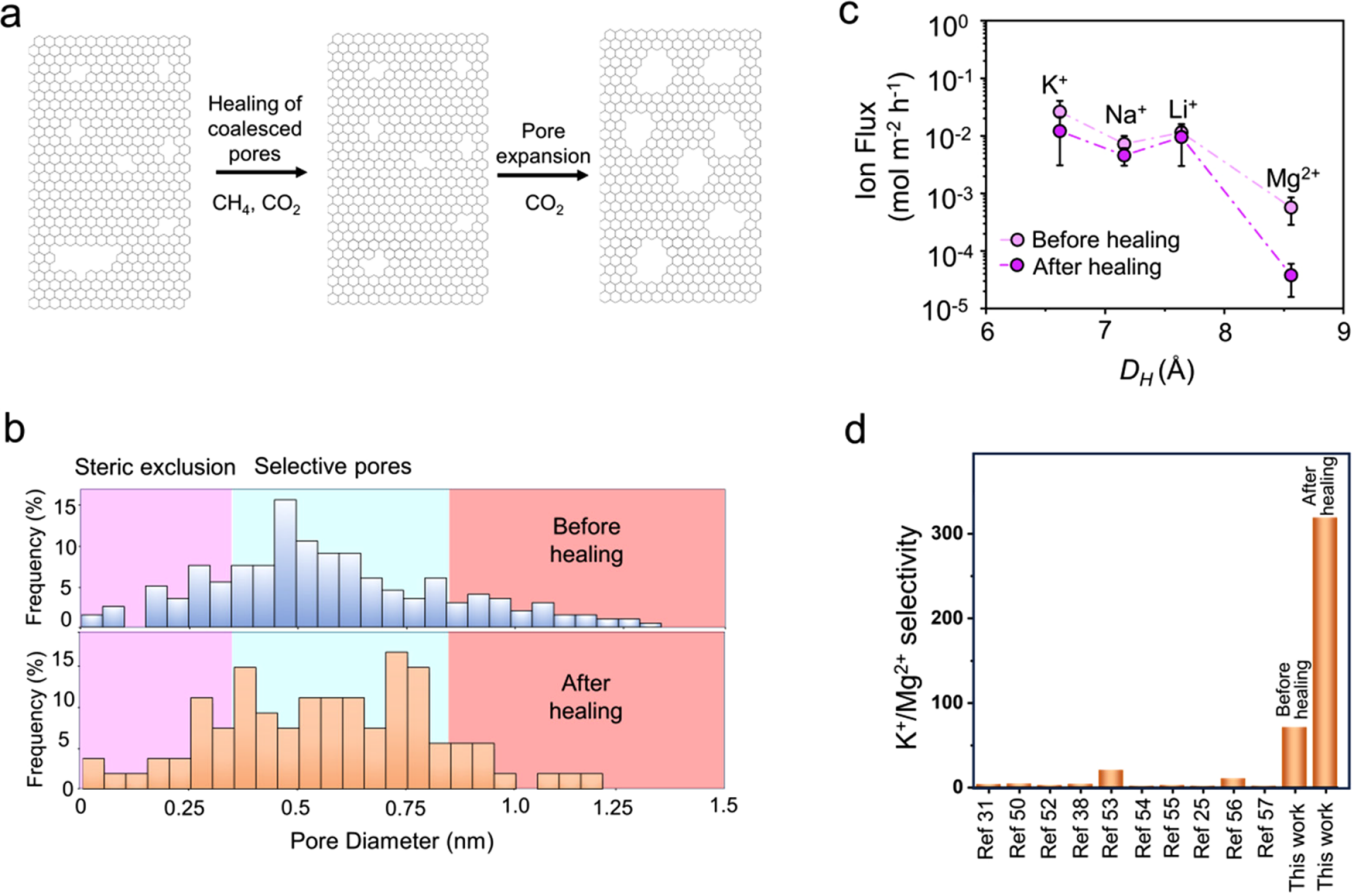
Healing of pores larger than 10 Å. (a) Schematic illustrations of the healing approach involving exposing as-synthesized PG_3–4Å_ to CH_4_/CO_2_ environment followed by CO_2_ expansion. (b) Comparison of pore size distribution obtained after the healing approach with the control sample. (c) Diffusive ion flux from PG_<8.5Å_ before and after healing. (d) Comparison of K^+^/Mg^2+^ selectivities from this study to the literature (also listed in Table S3) on ion transport from two-dimensional materials.

Motivated by the role of Å-scale pores in promoting ion–ion selectivity via the partial dehydration effect, we developed a pore size tuning approach that decreases the fraction of pores larger than 10 Å generated from the pore–pore coalescence, as discussed before. Briefly, we developed a healing approach for as-synthesized porous graphene (PG_3–4Å_), resting on the Cu foil, to shrink coalesced pores. This was achieved by creating a CVD-like environment in the presence of CH_4_ and CO_2_ (Figure 4a). Pores expose underlying catalytic Cu to CH_4_ which then onsets graphene growth events leading to pore shrinkage (Figure S19, Supplementary Note S5). The presence of CO_2_ onsets a competition between CH_4_-aided crystallization of graphene domains starting from the pore edge and CO_2_-aided pore edge expansion.^54^ Indeed, we observed that the presence of CO_2_ limits graphene crystallization in the CVD environment (Figures S20 and S21), ensuring that pores are not completely closed.

The tuning of the pore size distribution by our healing approach is confirmed by the comparison of the distribution in the samples before and after the healing step (Figure 4b). We observed a significant decrease in the population of pores larger than 10 Å. The population of nonselective pores larger than 10 Å decreased by half (Figure 4b). This led to improved selectivities with K^+^/Mg^2+^ selectivities of 255 and 345, and Li^+^/Mg^2+^ selectivities of 220 and 264 from two samples, respectively (Figure 4c). We compared the ion–ion selectivity data from graphene pores in this study with those from two-dimensional materials in the literature^8,9,35,37,55−60^ (Figure 4d, Table S3). The comparison highlights that ion–ion selectivity from porous graphene is significantly high, which we attribute to tight control of the size distribution of pores in this study.

## Conclusion

Overall, we present a route to prepare crack-free centimeter-scale graphene film for ion–ion separation, and we demonstrate that porous graphene can be synthesized with pore size below 8.5 Å by controlled pore expansion and healing of coalesced pores. Such pores are extremely interesting for studying ion transport mainly because ion confinement in these pores leads to partial dehydration of the hydration shell which then dictates the overall ion mobility across the pore. This resulted in the observation of record-high ion–ion selectivity from zero-dimensional pores. The fact that the pore size distribution can be tuned in the Å regime makes porous graphene a highly interesting platform for exploring and manipulating the transport of similar-sized ions, e.g., by functionalization of the graphene edge mimicking functional groups in the protein channels. This report will inspire the exploration of zero-dimensional pores, such as those in porous graphene, for other highly sought-after nanofluidic applications, e.g., phase transition and reaction under two-dimensional confinement.

## Experimental Section/Methods

### Synthesis of G_ID_

Single-layer graphene was synthesized by the CVD using a preannealed copper foil (50 μm-thick, 99.9% purity, Strem) as reported before.^61^ Briefly, the annealed copper foil was exposed to a CO_2_ and H_2_ atmosphere at 1000 °C for 30 min, respectively. Subsequently, 24 sccm of CH_4_ and 8 sccm of H_2_ were introduced into the reactor at a total pressure of 460 mTorr for 30 min. The reactor was then cooled to room temperature to obtain G_ID_.

### Synthesis of PG_3–4Å_

A homemade setup was used for incorporating pores into the graphene lattice. The setup was composed of a heated reactor chamber with a thermocouple placed inside the heating zone. Briefly, as-synthesized G_ID_ resting on Cu was placed inside the reactor under a continuous Ar flow (100 sccm, 1 bar) at 43 °C. After the whole system reached thermal equilibrium, the Ar flow was cut off, followed by the injection of an O_3_/O_2_ mixture (9% O_3_ on a molar basis, Atlas 30, Absolute Ozone) into the chamber for 1 h. Thereafter, heating was turned off, and the O_3_ was purged by Ar flow to obtain PG_3–4Å_.

### Pore Expansion by CO_2_ for the Synthesis of PG_<8.5Å_ and PG_>10Å_

For pore expansion by CO_2_, the as-obtained PG_3–4Å_ was placed in a CVD furnace and was exposed to H_2_ flow (100 sccm) to purge out air. Next, the sample was heated up to 800 °C. Subsequently, a mixture of H_2_ (23 sccm) and CO_2_ (50 sccm) was introduced for 5 min of pore expansion reaction. The reactor was then cooled down to room temperature. For the synthesis of PG_>10Å_, the mixture of H_2_ and CO_2_ was introduced for 8 min. All subsequent steps were the same as those for PG_<8.5Å_.

### Healing of Large Coalesced Pores

For the healing of large pores, prior to the CO_2_ expansion step, PG_3–4Å_ resting on a Cu foil was exposed to a mixture of CH_4_ and CO_2_ (molar ratio 2:1) with H_2_ as a protective gas at 800 °C for 10 min. The CO_2_ expansion was continued as above, i.e., by flowing a mixture of H_2_ (23 sccm) and CO_2_ (50 sccm) for 5 min.

### Fabrication of the Self-Supporting Graphene Membrane

The as-obtained graphene sample on the copper foil was reinforced by a two-layer composite film composed of a nanoporous carbon (NPC) film^48^ and a Nafion film. First, the fabrication of the NPC on graphene was performed using a method reported elsewhere^48^ with some minor modifications. Briefly, the precursor of NPC was prepared by dissolving 0.4 g of poly(styrene-*b*-4-vinylpyridine) and 0.8 g of turanose in 4 g of N,N-dimethylformamide. Subsequently, the solution was heated to 180 °C for 3–5 h. The NPC film was obtained by spin coating the as-obtained solution on the porous graphene on the Cu foil, followed by the pyrolysis at 500 °C in a H_2_/Ar atmosphere for 1 h. The NPC/Graphene/Cu was then spin-coated by a Nafion solution (Nafion D-520 dispersion, 5% w/w in water and 1-propanol) and the Nafion was cross-linked at 120 °C for 1 h to obtain Nafion/NPC/Graphene/Cu. The Cu foil was etched by FeCl_3_, and the Nafion/NPC/Graphene membrane was floated in HCl and deionized (DI) water for rinsing. Finally, the floating graphene film was scooped on a polytetrafluoroethylene (PTFE) annular disk with hole size of 0.8–2 cm.

### Ion Diffusion Tests

A homemade diffusion cell with two 50 mL reservoirs was used for studying ion diffusion. The self-supporting graphene membrane on PTFE annular disks was sandwiched by another PTFE annular disk and was mounted between two reservoirs, with the graphene side toward the feed reservoir. The diameter of the effective area is 0.8 cm, which corresponds to the effective area of 0.5 cm^2^. The feed solution contained salt solutions (KCl, NaCl, LiCl, MgCl_2_, >98%, Sigma-Aldrich). The permeate side contained Milli-Q water. During the measurement, the concentration changes in the permeate side were negligible compared to the feed concentration; therefore, the driving force for ion diffusion remained constant. For the single-ion tests of Na^+^, Li^+^, K^+^, and Mg^2+^, a precalibrated conductivity probe (Mettler-Toledo GmbH, SevenCompact Cond. Meter S230) was placed in the permeate chamber to record the conductivity increase. The conductivity (μS/cm) was converted to concentration (mol/L) by obtaining a calibration curve of conductivity as a function of concentration by inductively coupled plasma–optical emission spectrometry (ICP-OES, 5110, Agilent, Figure S7). For the proton test, the calibration curve is calibrated by a pH meter (Figure S8). For mixed-ion tests (Figure S11) and the measurement of Zn^2+^, Fe^3+^, and Al^3+^, the concentration of each cation was measured by ICP-OES as a function of time (Figure S22). The cation flux, *J*, was then calculated as
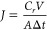
2where *C*_*r*_ is the concentration in the permeate chamber, *V* is the volume of the chamber, *A* is the area of the graphene film, and Δ*t* is the time.

The selectivity, *S*_*ij*_, of one cation, *i*, over another one, *j*, was calculated as
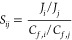
3where *J*_*i*_ is the flux of cation *i* and *C*_*f*,*i*_ is the feed concentration of cation *i*.

### Raman Characterizations and Isotope Labeling

Raman spectroscopy was done by a Renishaw inVia confocal spectroscope equipped with a 457 nm excitation laser and ZEISS Plan-Apochromat 63X/1.4 Oil DIC objective. The combination of 457 nm laser and ZEISS Plan-Apochromat 63X/1.4 Oil DIC objective resulted in a spatial resolution of 200 nm, calculated by the 0.61λ/NA equation, where λ is the wavelength of the laser and NA is the numerical aperture of the objective. The laser power was kept below 1 mW to prevent damaging the samples due to localized heating. Samples were first transferred onto SiO_2_/Si substrates using the common PMMA transfer method. Briefly, a 950 PMMA solution in anisole from Microchem was spin-coated on the sample at 1000 and 2000 rpm for 1 min each. Then, the reaction mixture was heated at 70 °C for 30 min. After etching the Cu foil by 0.5 M Na_2_SO_4_ solution and rinsing the PMMA/graphene film with DI water, the floating film was picked up by an O_2_-plasma-treated SiO_2_/Si substrate. The sample was dried in air overnight, and it was heated at 150 and 190 °C for 10 min each before removing the PMMA layer with acetone. More than 10000 spectra were collected for each map. The 2D peak intensity and *I*_*D*_/*I*_*G*_ and *I*_2*D*_/*I*_*G*_ ratios were calculated by subtracting the background and curve fitting the 2D, G, and D peaks by a custom program in MATLAB. The G peak around ∼1580 cm^–1^ represents the symmetrical opposing motion of carbon atoms in a single direction within the plane of the graphene sheet. The D peak at around 1350 cm^–1^ represents a symmetry breaking “defect” bonded to the six-membered carbon ring. This is commonly used to probe functionalization, sheet size, and defect density by crudely correlating them with peak ratio *I_D_*/*I_G_*. The 2D peak around 2700 cm^–1^ represents the double- or triple-resonance condition of this interaction. Commonly, *I_2D_*/*I_G_* is extracted and used as a measure of the number of graphene layers.

### AC-HRTEM Imaging

AC-HRTEM imaging was carried out using Thermo Fisher Scientific Titan Themis^3^ operated at 80 kV. The microscope is equipped with a monochromated high-brightness field emission gun (X-FEG), an image aberration corrector (CEOS CETCOR), and a 4K-resolution complementary metal-oxide-semiconductor (CMOS) camera (ThermoFisher Ceta). The sample preparation followed our previous method^48^ using paraffin to transfer the graphene sample onto the SiN TEM grid. The TEM grids with freestanding graphene were treated by exactly the same procedure to obtain the samples for TEM imaging. Images were captured by using an exposure time of 200 ms and a camera binning of 4. The dose rate during imaging ranged from 1.2–1.4 × 10^4^ e^–^ Å^–2^ s^–1^. Final images were obtained by integrating 10 to 20 consecutive images and denoised by using high pass and Gaussian blur filters. The pore density is calculated by dividing the number of pores by the area of the selected TEM image area.

### Other Characterizations

Scanning electron microscope (SEM) images were obtained by using an FEI Teneo scanning electron microscope at 1.0 kV and working distances of 2.5–5.0 mm. No conductive coating was applied to the substrates before the SEM imaging. X-ray photoelectron spectroscopy (XPS) measurements on graphene resting on the Cu foil were carried out on Axis Supra (Kratos Analytical) using the monochromated Kα X-ray line of an aluminum anode. The pass energy was set to 20 eV, and the step size was set to 0.1 eV. The peak fitting was performed by using CasaXPS, and the Shirley method was used for background subtraction.

## Supporting Information Available

Further data including detailed description of pore diameter measurement, ion diffusion test, XPS analysis, MD simulation, and pore shrinking strategy and figures showing the Raman spectrum, mechanical property of the membrane, calibration curve, XPS spectrum, and performance comparison (PDF)
