# Supplementary material for: Tuning Pore Size in Graphene in the Angstrom Regime for Highly Selective Ion–Ion Separation
Source: ACS Nano. 2024 Feb 6;18(7):5571–80. doi: 10.1021/acsnano.3c11068 (PMC10883049; doi:10.1021/acsnano.3c11068)
Supplement: Supplementary file 1 — nn3c11068_si_001.pdf [file nn3c11068_si_001.pdf]

Supporting Information

Tuning pore size in graphene in Å regime for highly selective ion-ion separation

*Kangning Zhao[1, #], Wan-Chi Lee[1, #], Mojtaba Rezaei [1, #], Heng-Yu Chi[1], Shaoxian Li[1], Luis Francisco Villalobos[1], Kuang-Jung Hsu[1], Yuyang Zhang[2], Feng-Chao Wang[2], Kumar Varoon Agrawal[1, *]*

[1] Laboratory of Advanced Separations (LAS), École Polytechnique Fédérale de Lausanne (EPFL), Sion, CH-1950 Switzerland

[2] CAS Key Laboratory of Mechanical Behavior and Design of Materials, Department of Modern Mechanics, University of Science and Technology of China, Hefei 230027, China

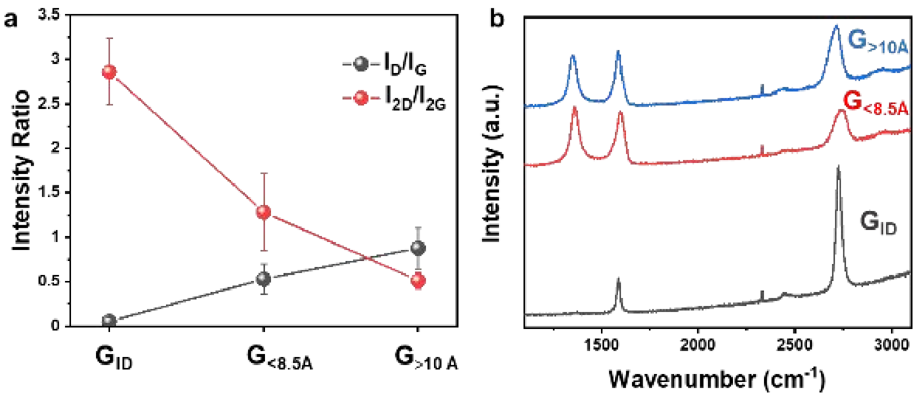

Figure S1. Raman spectrum of samples with different postsynthetic etching.

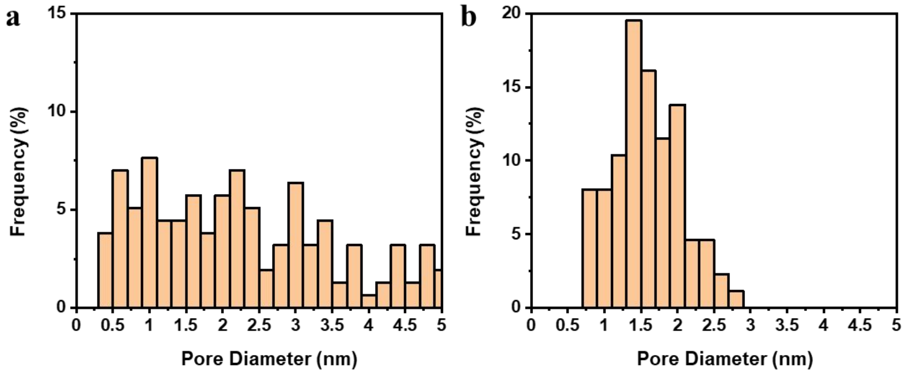

Figure S2. Formation of undesired large pores by a) pores expansion using O_3, and b) O_2 plasma (6 s).

Supplementary Note S1

The pore diameter was determined by a sphere fitting method. Briefly, a circle is fitted inside a pore by locating the edge carbon atoms, where we get the diameter D_I as shown in Figure S3. This circle touched the edge of the edge carbon atoms. Next, using the knowledge of the atomic radii of carbon atom, we drew another circle that intersects the center of these edge carbon atoms. The diameter of this circle is noted as D_C (Figure S3). Next, we subtracted the nonbonded interaction length between graphitic carbon and water molecule, $2^{1/6}$ σ (=3.86 Å) corresponding to the position of the potential well[1], from D_C to determine the effective pore diameter. This is also consistent with the pore diameter used in the MD simulations in this study.

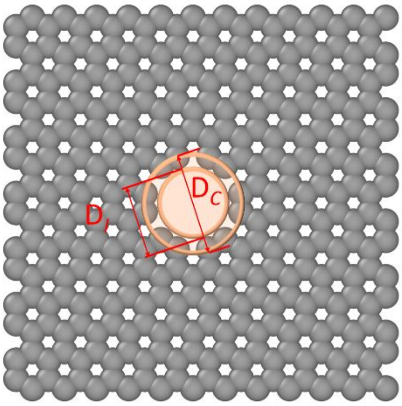

Figure S3. Schematic showing the measurement of the pore size.

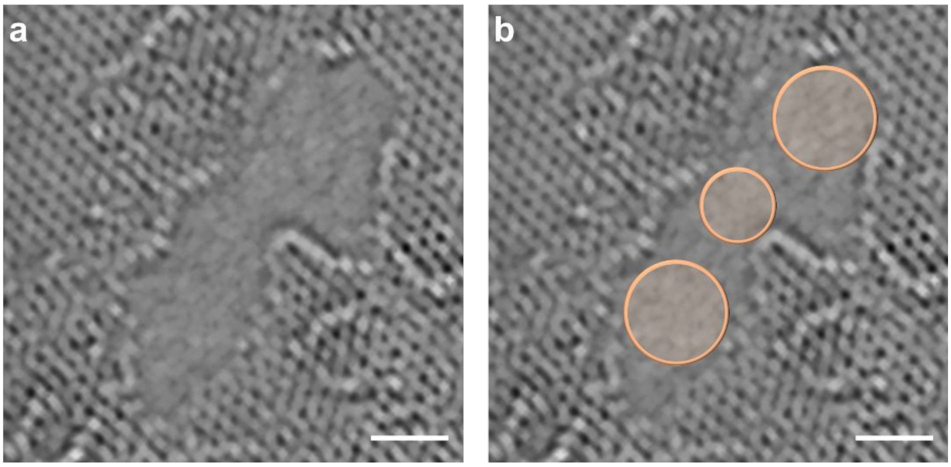

Figure S4. a) AC-HRTEM image of an elongated pore. b) One could fit three individual pores in this enlarged pore which illustrates that this elongated pore is likely created by pore-pore coalescence. Scale bars represent 1 nm.

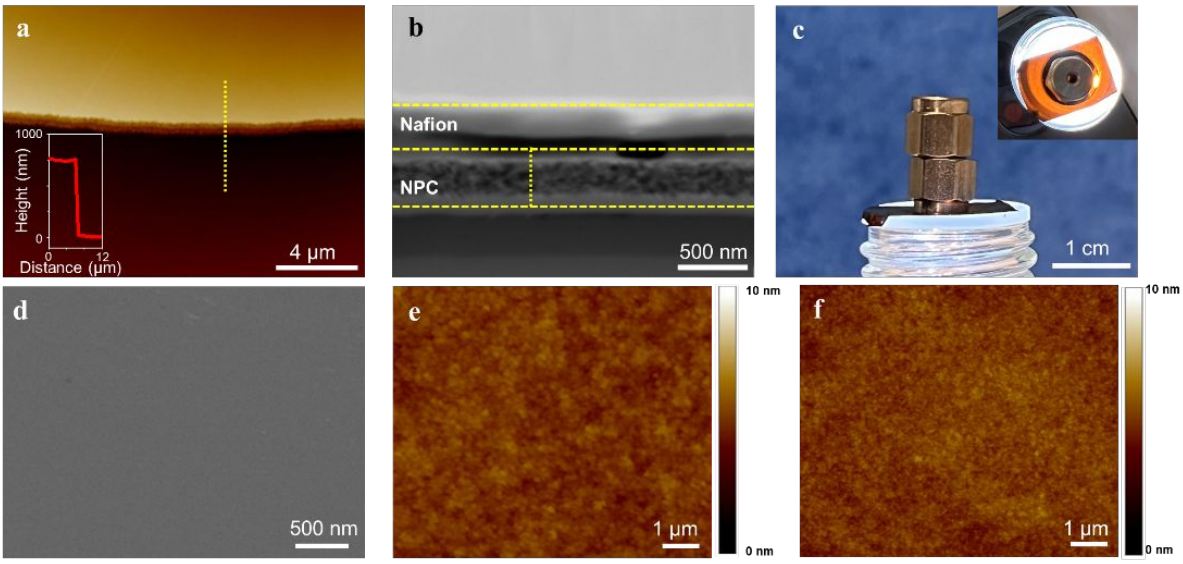

Figure S5. a) AFM image of the reinforced graphene revealing the thickness of the support film. b) Cross-sectional SEM image of the support film. c) Suspended graphene on 1-cm-sized annular disk loaded with 7 g weight. The inset shows the top view. d) Top-view SEM image of graphene reinforced with the support film. AFM image of the sample with graphene side in (e) and Nafion side in (f).

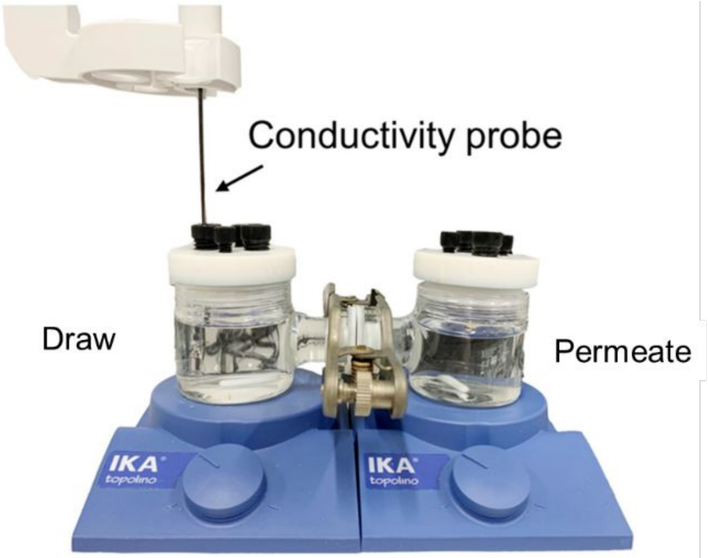

Figure S6. Picture of the diffusion cell used in this study.

Supplementary Note S2

In the H-cell, the ion transport is via diffusion driven by concentration difference of ions across porous graphene in the two well-mixed reservoirs. In the process, the osmotic pressure unavoidably drives water to diffuse from the dilute salt solution to the concentrated salt solution, in a opposite direction to the ion transport[2]. We note that the recorded water permeance of porous graphene resting on the support film was very low (0.0016 $L\ m^{-2}\ h^{-1}\ bar^{-1}$) due to the resistance from the Nafion layer in the support film. The ultralow water permeance does not affect our measurements since each single ion test takes about 6 h and the water transport can be ignored. Nafion layer served a dual role, (i) to improve the mechanical robustness of the graphene film, and (ii) to reduce the water crossover from the permeate side to feed side by forward osmosis, given that the feed side had ion solution with concentration of 1 M. The second aspect relates to slow diffusivity of free water through the Nafion layer.

We also note that Nafion support film does not dominate the ion transport through membrane because the ion flux through porous graphene is significantly lower than that from the support film.

We also note that the as-prepared porous graphene has a high pore density of $2.2 \times 10^{12}\ cm^{-2}$, with a small distance between each pore in the range of 1-16 nm which should eliminate the perforated screen effect[3].

We are aware of the presence of thick unstirred layer in the vicinity of porous graphene which could control mass transfer to a certain extent. The presence of an unstirred layer decreases the ion flux as well as ion selectivity. Therefore, porous membrane would demonstrate even higher performance in terms of ion flux and selectivity if the system is optimized to decrease the unstirred layer, e.g., through cross flow condition.

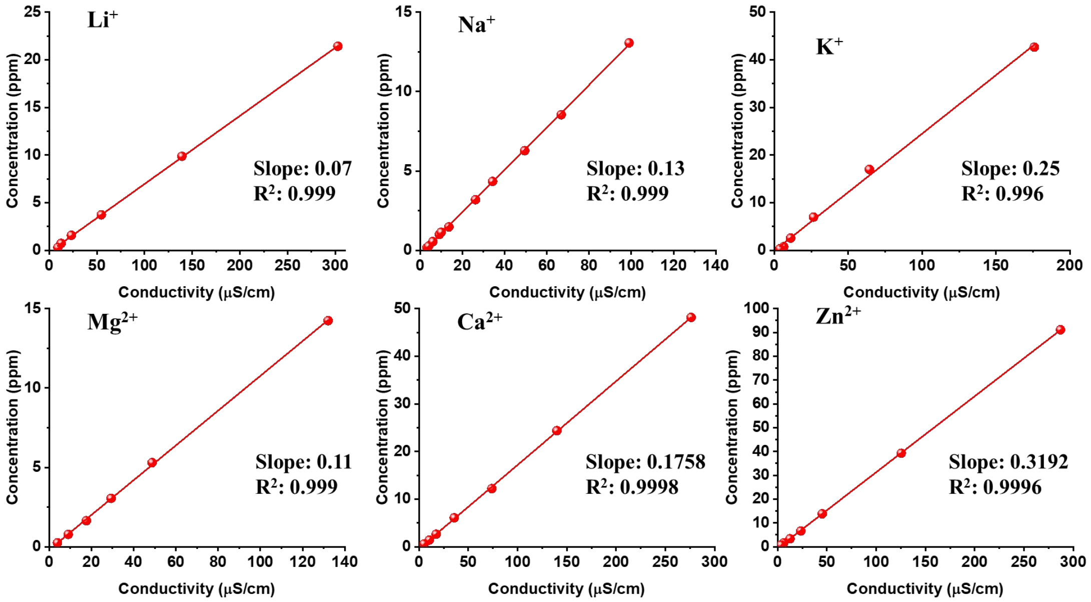

Figure S7. ICP based calibration curves for Li^+, Na^+, K^+, Mg^{2+}, Ca^{2+}, and Zn^{2+}.

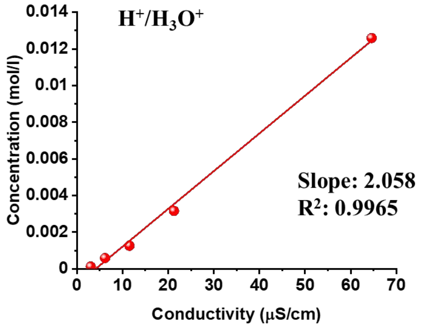

Figure S8. Calibration curve for proton using the pH meter.

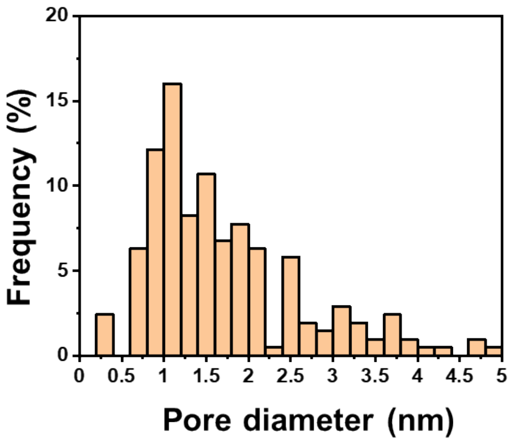

Figure S9. Pore size distribution for the sample $PG_{>10Å}$.

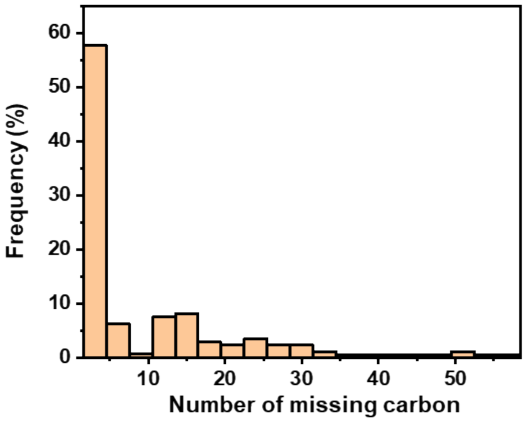

Figure S10. Pore size distribution of $PG_{3\text{-}4Å}$ in terms of number of missing carbon atoms.

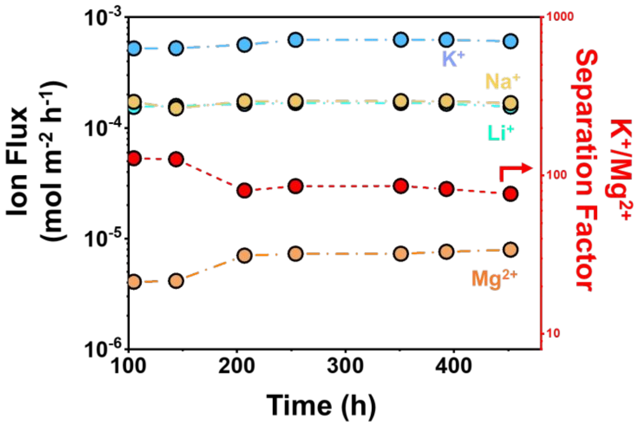

Figure S11. Long-term ion diffusion data from $PG_{<8.5Å}$ using an equimolar ion mixture (0.1 M of KCl, NaCl, LiCl, and $MgCl_2$).

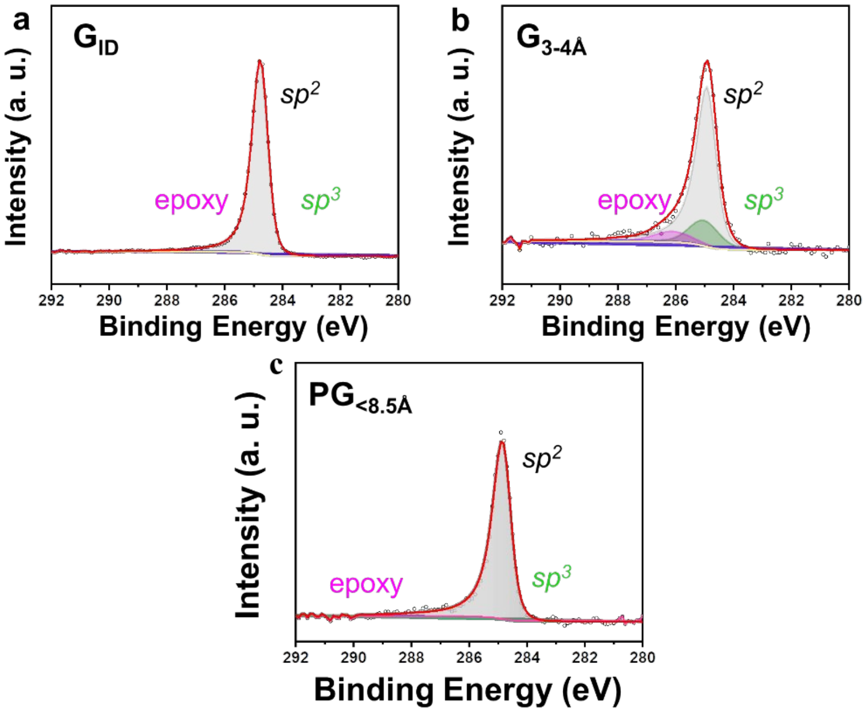

Figure S12. C1s XPS spectra from a) G_{ID}, b) $PG_{3\text{-}4Å}$ and c) $PG_{<8.5Å}$.

Supplementary Note S3

To understand the presence of charge on graphene pores, we carried out X-ray photoelectron spectroscopy (XPS) to probe O-functional group on the surface. As a control, G_{ID} showed negligible O concentration, consistent with the fact that graphene lattice is composed of sp^2 carbon (Figure S12a). The C1s XPS spectrum from $PG_{3\text{-}4Å}$ shows an obvious shoulder at 286.1 eV corresponding to the presence of O-functional groups. However, the O-functional groups are absent in the graphene sample after subsequent CO_2-led pore expansion. This is mainly because the O-functional group from O_3-treatment are generated in the form of O-clusters surrounding the pore[4] which are then gasified during CO_2 expansion. Among the trace functional group, we could identify semiquinone groups (0.39%) which is expected at the pore edge, and ether groups (2.29%), however, their concentrations were similar to that in G_{ID}, indicating their contribution is likely from unavoidable atmospheric contamination (Table S1).

Table S1. Summary of the fitting result of XPS spectrum in Figure S14

	C *sp*^{2}	C *sp*^{3}	semiquinone	epoxy
G_{ID}	99.99%	7.93%	0.41%	3.5%
$PG_{3\text{-}4Å}$	78.22%	14.36%	0.24%	7.18%
$PG_{<8.5Å}$	99.75%	0%	0.25%	0%

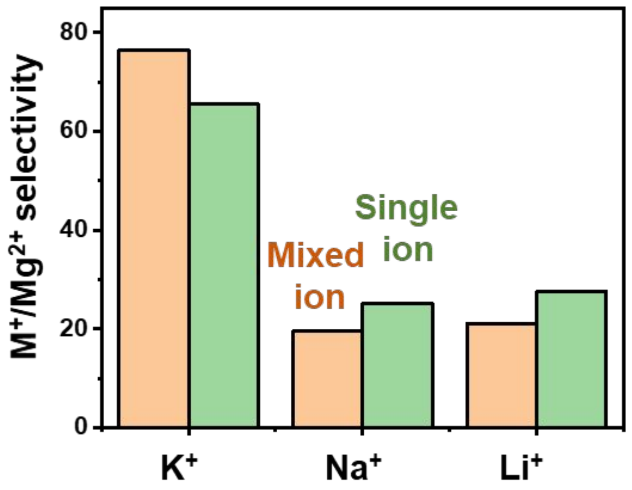

Figure S13. Comparison of the ion selectivity in single ion and mixed ion test. Here M^+ refers to either K^+, Na^+ or Li^+.

Supplementary Note S4
Simulation Models and Methodology

To compare the hydration shells of ions in the bulk solution and in a confined nanopore, two kinds of models were built. The first model is a cubic simulation box with a size of 30 Å × 30 Å × 30 Å, which contains 900 water molecules and one cation ion (Mg^{2+} or K^+), as shown in Figure S14a. In the second model, a monolayer graphene was inserted into the simulation box, which is parallel to the XY plane, as shown in Figure S14b. The size of the second simulation box was adjusted slightly to accommodate the lattice structure of the graphene.

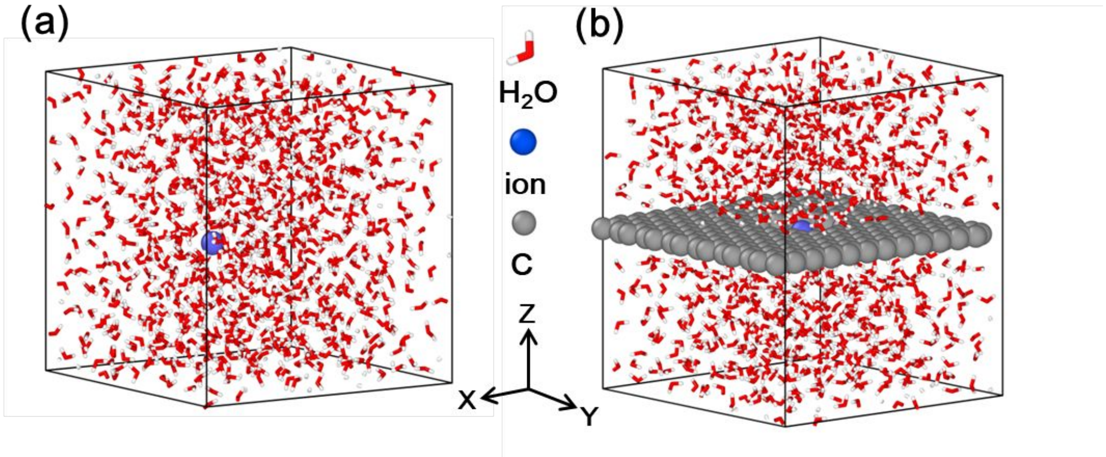

Figure S14. MD simulation models. a) Ion and its bulk solution. b) Ion restricted in graphene pore.

The pores were constructed by removing some carbon atoms (12, 24, 36, 42, 54, and 60 carbon missing) from the graphene sheet. After subtracting the carbon atoms, the pore diameter is estimated by fitting the inscribed circle along the center of the edge carbon atom. Considering the carbon-water interaction, the diameter of the accessible pore is determined by subtracting the distance of the graphitic carbon–water nonbonded interaction length ($\sqrt[6]{2}\,\sigma_{C-O_w} = 3.857$Å). Thus, the pore diameters of pores range from 3.7 Å to 12.0 Å. During the simulations, the graphene sheet was set to be rigid and fixed at its initial position (Z = 0). The cation was restricted at the pore along Z axis it was allowed to move in the other two directions. Interactions in the simulation models were described by the standard 12-6 Lennard-Jones potential together with a Coulombic term. The potential energy was calculated in the form of

$$U(r) = 4\varepsilon\left[\left(\frac{\sigma}{r}\right)^{12} - \left(\frac{\sigma}{r}\right)^{6}\right] + \frac{Cq_1q_2}{\epsilon r}, \qquad \text{(S1)}$$

where r is the distance between two atoms, σ and ε are the force field parameters characterize interactions between different atoms, C is the energy constant, ϵ is the dielectric constant and

q refers to the atom charge. The simple point charge extended (SPC/E) model[5] was used to describe water molecules. The force field parameters for interactions between atoms of the same type are listed in Table S2.[6] Parameters for the graphene-water interactions were taken from previous literature[1]. The Lorentz–Berthelot combination rules were used to obtain parameters for interactions between different species. The short-range interactions were truncated at a cutoff distance of 12 Å, and the long-range Coulomb interactions were computed by utilizing the particle–particle particle–mesh (PPPM) algorithm[7]. Periodic boundary conditions are applied to all three directions.

Table S2. Force field parameters[6]

	σ (Å)	ε (eV)	q (e)
K^+	2.838	0.0186	+1.0
Mg^{2+}	2.546	0.0009	+2.0
H	0	0	+0.4238
O	3.166	0.0067	–0.8476
C	3.390	0.0030	0

After the initial energy minimization, each system was first equilibrated in the canonical ensemble for 0.1 ns with the timestep of 1 fs. The temperature of the system was maintained at 300 K using a Nose-Hoover thermostat. Then the simulations are performed for another 5 ns to collect the data for further analysis. All the MD simulations were carried out using LAMMPS[8].

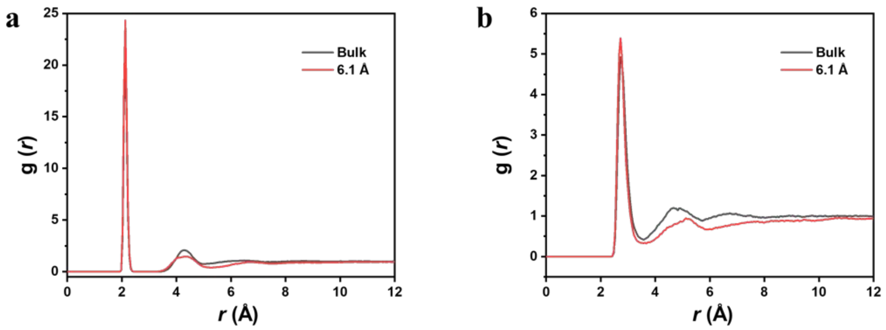

Figure S15. Radial distribution function $g(r)$ for (a) Mg^{2+} and (b) K^+ in the bulk and around a 6.1 Å pore.

The ion-oxygen radial distribution function (RDF) of Mg^{2+} and K^+ are shown in Figure S15. The ion-oxygen RDF of ions in bulk solution and in nanopores with the diameter of 6.1 Å are

plotted. The curve shows the density distribution of oxygen atom around the ion, in which the peaks indicate the existence of hydration shells.

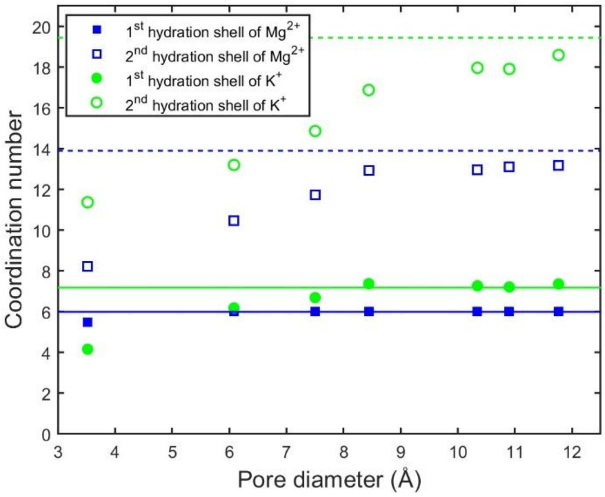

Figure S16. Coordination number of water molecules in the first and second hydration shells of Mg^{2+} or K^+ as a function of pore diameter. The solid lines refer to the bulk values of the coordination number of ions' first hydration shell, and the dashed lines correspond to ions' second hydration shell.

The coordination number of ions as a function of pore size is shown in Figure S16. The coordination number gives the number of water molecule in the ion's hydration shell. In Figure S16, the solid lines refer to the bulk values of the coordination number of ions' first hydration shell, and the dashed lines correspond to ions' second hydration shell. We found that no dehydration events occur in the first hydration shell of Mg^{2+} when it goes through nanopores whose diameter is larger than 6.1 Å. Evident dehydration happens in the second hydration shell of both ions and first hydration shell of Na^+. We estimated the energy penalty associated with water loss, ΔE, as following:

$$\Delta E = \delta N_1 * E_1 + \delta N_2 * E_2 \quad \text{(S2)}$$

where δN_1 and δN_2 refer to loss in coordination number in first and second shell, respectively. E_1 and δE_2 refer to the interaction energy between ion and water molecule in first and second shell, respectively (Figure 3h). The resulting values of ΔE for K^+ and Mg^{2+} are compared in Figure 3j indicating increase in free energy of 0.6 and 1.0 eV, respectively.

Supplementary Note S5

Pore shrinkage in the presence of CH_4

We verified the growth mechanism through the combination of carbon isotope labelling and Raman spectroscopy mapping. For this, first, G_{ID} resting on Cu foil was exposed to CO_2 at 800 ºC to expand intrinsic vacancy defects in micron-sized pores. The motivation behind generation of micron-sized pores is that these large pores can be easily visualized (Figure S17). These pores then become the starting point to study their shrinkage.

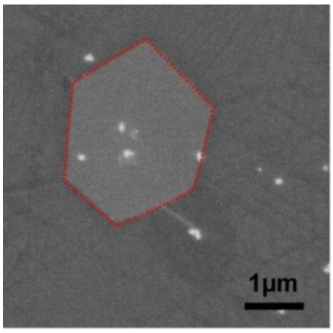

Figure S17. A CO_2-expanded micron size pore in graphene.

We then carried out an experiment where micron-sized graphene pore was exposed to ^{13}C CH_4 at 800 ºC. This was motivated by the fact that Raman spectroscopy can distinguish graphene composed of ^{12}C versus ^{13}C, attributing to the red shift of the phonon energy (wavenumber in Raman spectroscopy)[9] due to increased mass of ^{13}C. Figure S18 illustrates this where a difference of 110 cm^{-1} in the 2D peak position was observed (2D peak at 2725 cm^{-1} for ^{12}C and 2615 cm^{-1} for ^{13}C). Similarly, G peak position also goes through a shift. Therefore, shift in 2D or G peak positions can be used to track the precursor contributing to graphene growth.

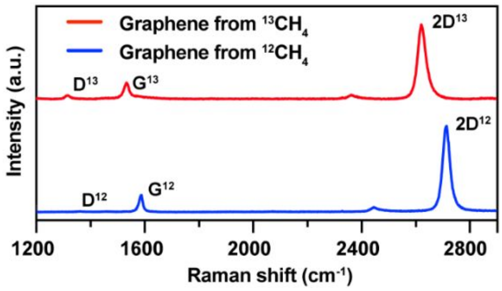

Figure S18. Raman spectra of graphene grown from $^{12}CH_4$ and $^{13}CH_4$.

In the next experiment, G_{ID} was synthesized by ^{12}C CH_4, micron-sized pores were created by CO_2, following which ^{13}C CH_4 exposed to shrink the pore. The Raman mapping of resulting pore is shown in Figure S19. Mapping and of $2D^{12}$ and $2D^{13}$ peak intensity clearly revealed shrinkage of the pore. The graphene area around the pore yielded 2D peak position from ^{12}C, representing as-synthesized graphene, while the newly grown graphene domains inside the pore were derived from ^{13}C. We observed growth in the core of the pore as well as pore

shrinkage from the pore edge. Figure S19d shows the acquired spectra from various points (marked by the numbers 1 – 6 on Figure S19c) in and around the pore. Y-axis scale for left (containing D and G peaks) and right (containing 2D peak) halves of the figure are different for better readability. Each side of the figure is normalized by the highest observed intensity (belonging to spectrum 6). Spectrum 1 represents the newly added graphene domain by ^{13}C CH_4 and matched the Raman spectrum of the ^{13}C-grown graphene in Figure S19. On the contrary, spectrum 6 that was acquired from outside the pore displayed the ^{12}C-grown SLG characteristics. This study concludes that the carbon precursor solely arrived from ^{13}C source from the pore healing experiment. This also rules out carbon contamination in the CVD reactor leading to the growth.

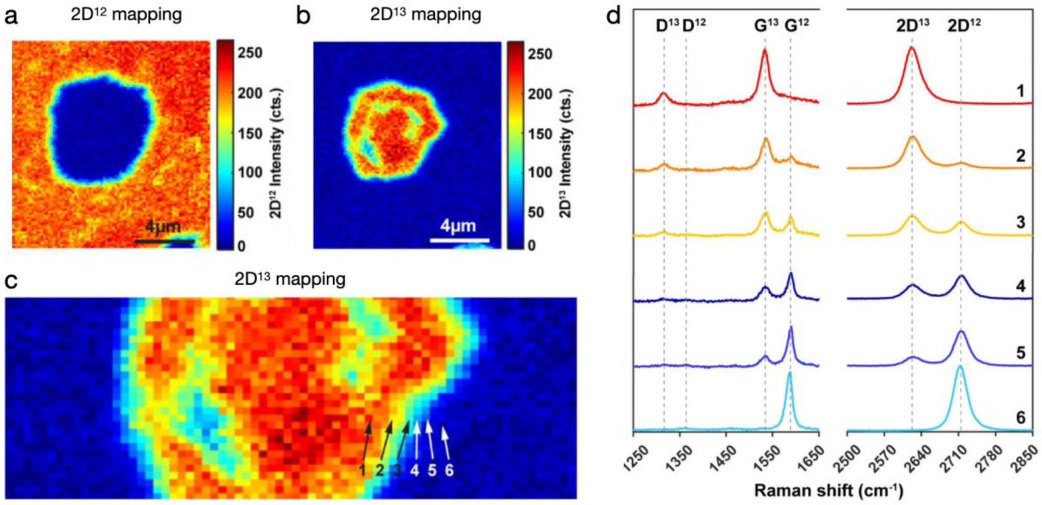

Figure S19. Raman map of 2D peak intensity from porous graphene subjected to pore shrinkage conditions involving exposed to ^{13}C CH_4. 2D peak mapping corresponding to ^{12}C (a) and ^{13}C (b). Enlarged map of (b) is shown in (c) and the corresponding Raman spectra of the points marked by numbers 1 – 6 are shown in (d).

Next, for pore shrinkage, we also added CO_2 in the presence of ^{13}C CH_4. Our hypothesis is that it onsets a competition between CH_4-aided crystallization of graphene domains starting from the pore edge and CO_2-aided pore edge expansion, based on a recent kinetic Monte Carlo simulation[10]. For example, while in the absence of CO_2, we observed growth of new graphene domains in the core of the pore (Figure S19), such domains were not observed in the presence of CO_2 (Figure S29). We only observed pore shrinkage from the edge of graphene. We attribute this to the etching of any nuclei-forming carbon precursors by CO_2.

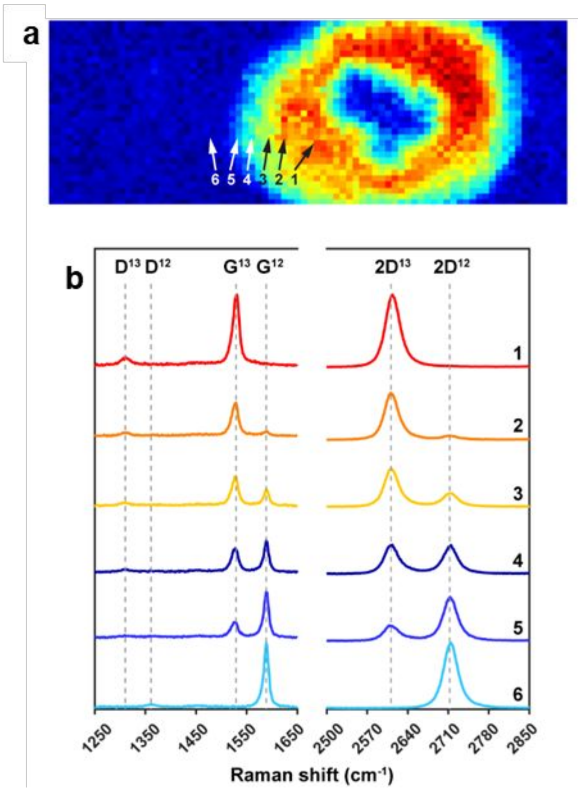

Figure S20. Raman map of 2D peak intensity from porous graphene subjected to pore shrinkage conditions involving exposure to a mixture of ^{13}C CH_4 and CO_2. a) 2D peak mapping corresponding to ^{13}C. b) Corresponding Raman spectra of the points marked by numbers 1 – 6.

Another evidence on the competitive roles of CO_2 and CH_4 comes from measuring the shrinkage rate of pores in the presence of varying ratio between CH_4 and CO_2. Figure S21 clearly shows that the pore shrinkage slows down after introducing CO_2. Further, at the ratio of 0.5, we did not observe any pore shrinkage.

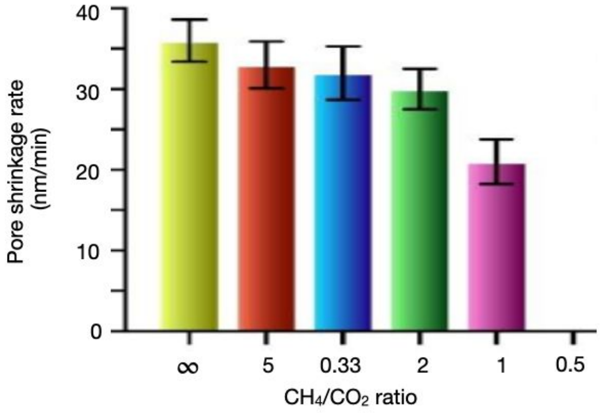

Figure S21. The observed pore shrinkage rate at various ratio of CH_4 and CO_2.

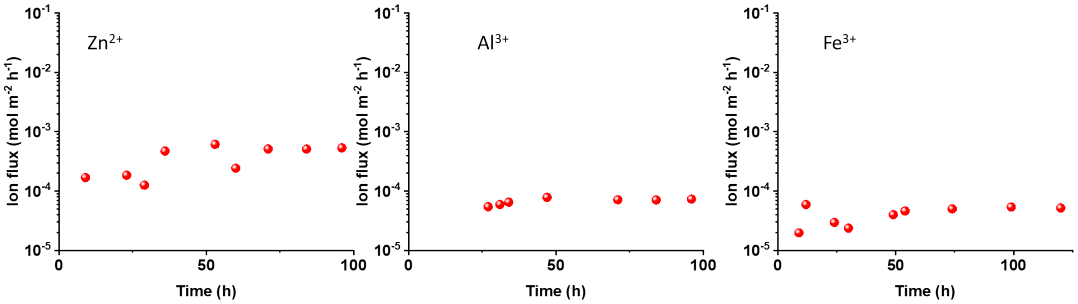

Figure S22. ICP curves of Zn^{2+}, Al^{3+}, and Fe^{3+} over time. The measurements were carried out because the hydrolysis effect of these ions is strong releasing a proton. Therefore, ion conductivity doesn't directly reflect ion concentration.

Table S3. Comparison of the ion-ion selectivity data from graphene pores in this study with those from two-dimensional materials in the literature.

**2D materials**	K^+/Mg^{2+} selectivity	Reference
Graphene with 20 nm pore	3.1	[11]
Graphene with 0.8 nm pore	4	[12]
Porous graphene	2.2	[13]
Graphene with 0.5 nm pore	3.5	[14]
Graphene with bubble	20	[15]
0.6-nm-sized MoS_2 pore	1.3	[16]
0.67-nm-sized graphite slit	2.3	[17]
Sub-manometer MoS_2 Pore	1	[18]
Porous graphene	10	[19]
Gated graphene	1	[20]
$PG_{<8.5Å}$ before healing	*70*	*This work*
$PG_{<8.5Å}$ after healing	*319*	*This work*

References

1. Wu, Y.; Aluru, N., Graphitic carbon–water nonbonded interaction parameters. *The Journal of physical chemistry B* **2013,** *117* (29), 8802-8813.
2. Wang, R.; Zhang, J.; Tang, C. Y.; Lin, S., Understanding selectivity in solute–solute separation: definitions, measurements, and comparability. *Environmental science & technology* **2022,** *56* (4), 2605-2616.
3. Yaroshchuk, A.; Bondarenko, M., Interaction of Potential Sources in Infinite 2D Arrays: Diffusion through Composite Membranes, Micro‐Electrochemistry, Entrance Resistance, and Other Examples. *Advanced Theory and Simulations* **2021,** *4* (11), 2100128.
4. Li, S.; Vahdat, M. T.; Huang, S.; Hsu, K.-J.; Rezaei, M.; Mensi, M.; Marzari, N.; Agrawal, K. V., Structure Evolution of Graphitic Surface upon Oxidation: Insights by Scanning Tunneling Microscopy. *Jacs Au* **2022,** *2* (3), 723-730.
5. Berendsen, H. J.; Grigera, J. R.; Straatsma, T. P., The missing term in effective pair potentials. *Journal of Physical Chemistry* **1987,** *91* (24), 6269-6271.
6. (a) Joung, I. S.; Cheatham III, T. E., Determination of alkali and halide monovalent ion parameters for use in explicitly solvated biomolecular simulations. *The Journal of physical chemistry B* **2008,** *112* (30), 9020-9041; (b) Li, P.; Merz Jr, K. M., Taking into

account the ion-induced dipole interaction in the nonbonded model of ions. *Journal of chemical theory and computation* **2014,** *10* (1), 289-297.
7. Hockney, R. W.; Eastwood, J. W., *Computer simulation using particles*. crc Press: 2021.
8. Plimpton, S., Fast parallel algorithms for short-range molecular dynamics. *Journal of computational physics* **1995,** *117* (1), 1-19.
9. Rümmeli, M. H.; Löffler, M.; Kramberger, C.; Simon, F.; Fülöp, F.; Jost, O.; Schönfelder, R.; Grüneis, A.; Gemming, T.; Pompe, W., Isotope-engineered single-wall carbon nanotubes; a key material for magnetic studies. *The Journal of Physical Chemistry C* **2007,** *111* (11), 4094-4098.
10. Dutta, S.; Vahdat, M. T.; Rezaei, M.; Agrawal, K. V., Crystallization of gas-selective nanoporous graphene by competitive etching and growth: a modeling study. *Scientific reports* **2019,** *9* (1), 5202.
11. Rollings, R. C.; Kuan, A. T.; Golovchenko, J. A., Ion selectivity of graphene nanopores. *Nature Communications* **2016,** *7* (1), 11408.
12. Fu, Y.; Su, S.; Zhang, N.; Wang, Y.; Guo, X.; Xue, J., Dehydration-determined ion selectivity of graphene subnanopores. *Acs Applied Materials & Interfaces* **2020,** *12* (21), 24281-24288.
13. Wang, H.; Su, L.; Yagmurcukardes, M.; Chen, J.; Jiang, Y.; Li, Z.; Quan, A.; Peeters, F. M.; Wang, C.; Geim, A. K., Blue energy conversion from holey-graphene-like membranes with a high density of subnanometer pores. *Nano Letters* **2020,** *20* (12), 8634-8639.
14. Jain, T.; Rasera, B. C.; Guerrero, R. J. S.; Boutilier, M. S.; O'hern, S. C.; Idrobo, J.-C.; Karnik, R., Heterogeneous sub-continuum ionic transport in statistically isolated graphene nanopores. *Nature nanotechnology* **2015,** *10* (12), 1053-1057.
15. Cantley, L.; Swett, J. L.; Lloyd, D.; Cullen, D. A.; Zhou, K.; Bedworth, P. V.; Heise, S.; Rondinone, A. J.; Xu, Z.; Sinton, S., Voltage gated inter-cation selective ion channels from graphene nanopores. *Nanoscale* **2019,** *11* (20), 9856-9861.
16. Feng, J.; Liu, K.; Graf, M.; Dumcenco, D.; Kis, A.; Di Ventra, M.; Radenovic, A., Observation of ionic Coulomb blockade in nanopores. *Nature materials* **2016,** *15* (8), 850-855.
17. Esfandiar, A.; Radha, B.; Wang, F.; Yang, Q.; Hu, S.; Garaj, S.; Nair, R. R.; Geim, A.; Gopinadhan, K., Size effect in ion transport through angstrom-scale slits. *Science* **2017,** *358* (6362), 511-513.
18. Thiruraman, J. P.; Masih Das, P.; Drndic, M., Stochastic ionic transport in single atomic zero-dimensional pores. *Acs Nano* **2020,** *14* (9), 11831-11845.
19. O'Hern, S. C.; Jang, D.; Bose, S.; Idrobo, J.-C.; Song, Y.; Laoui, T.; Kong, J.; Karnik, R., Nanofiltration across defect-sealed nanoporous monolayer graphene. *Nano Letters* **2015,** *15* (5), 3254-3260.
20. Wyss, R. M.; Tian, T.; Yazda, K.; Park, H. G.; Shih, C.-J., Macroscopic salt rejection through electrostatically gated nanoporous graphene. *Nano Letters* **2019,** *19* (9), 6400-6409.
